# Supplementary material for: Human coronavirus HKU1 recognition of the TMPRSS2 host receptor
Source: bioRxiv. 2024 Jan 9:2024.01.09.574565. Preprint. [Version 1] doi: 10.1101/2024.01.09.574565 (PMC10802434; doi:10.1101/2024.01.09.574565)
Supplement: 1 [file NIHPP2024.01.09.574565v1-supplement-1.pdf]

bioRxiv preprint doi: https://doi.org/10.1101/2024.01.09.574565; this version posted January 9, 2024. The copyright holder for this preprint (which was not certified by peer review) is the author/funder, who has granted bioRxiv a license to display the preprint in perpetuity. It is made available under aCC-BY 4.0 International license.

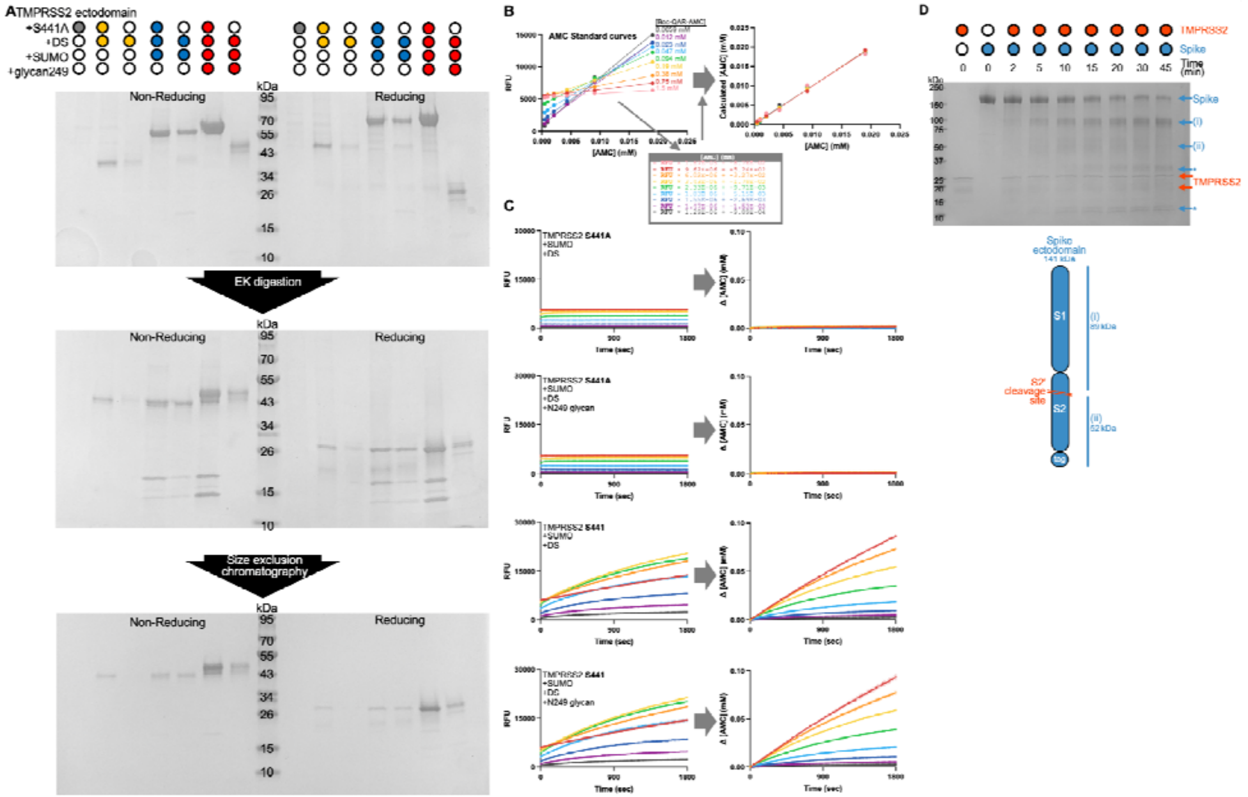

Figure S1. Design and functional characterization of TMPRSS2 ectodomain constructs. (A) Reducing and non-reducing SDS-PAGE analysis of the samples shown in Figure 1B, including the intermediate step between enterokinase (EK) digestion and size-exclusion chromatography (SEC) purification. (B) Summary of AMC standard curves used to calculate AMC release from the Boc-QAR-AMC peptide substrate accounting for the inner filter effect.

bioRxiv preprint doi: https://doi.org/10.1101/2024.01.09.574565; this version posted January 9, 2024. The copyright holder for this preprint (which was not certified by peer review) is the author/funder, who has granted bioRxiv a license to display the preprint in perpetuity. It is made available under aCC-BY 4.0 International license.

The left panel shows the relationship between AMC concentration and measured fluorescence (RFU). The lower inset shows the numerical equations of the linear relationships shown in the top panel. The right panel shows the calculated AMC concentration for each known AMC concentration shown in the left panel, using the equations in the lower inset at the indicated Boc-QAR-AMC concentrations. (C) Raw data and calculations used for Figure 1C; the color key is identical to Figure S1B. The left panels show the raw RFU over time for TMPRSS2 with and without the S441A mutation or the restored N249 glycan, while the right panel shows the change in calculated AMC concentration over time, using the equations from Figure S1B. (D) Reducing SDS-PAGE used for analysis shown in Figure 1D. Expected products (i) and (ii), based on TMPRSS2 cleavage at the S_2' site, are labeled, along with additional cleavage products (asterisks). Reaction progress was monitored by densitometry of the S peak only.

bioRxiv preprint doi: https://doi.org/10.1101/2024.01.09.574565; this version posted January 9, 2024. The copyright holder for this preprint (which was not certified by peer review) is the author/funder, who has granted bioRxiv a license to display the preprint in perpetuity. It is made available under aCC-BY 4.0 International license.

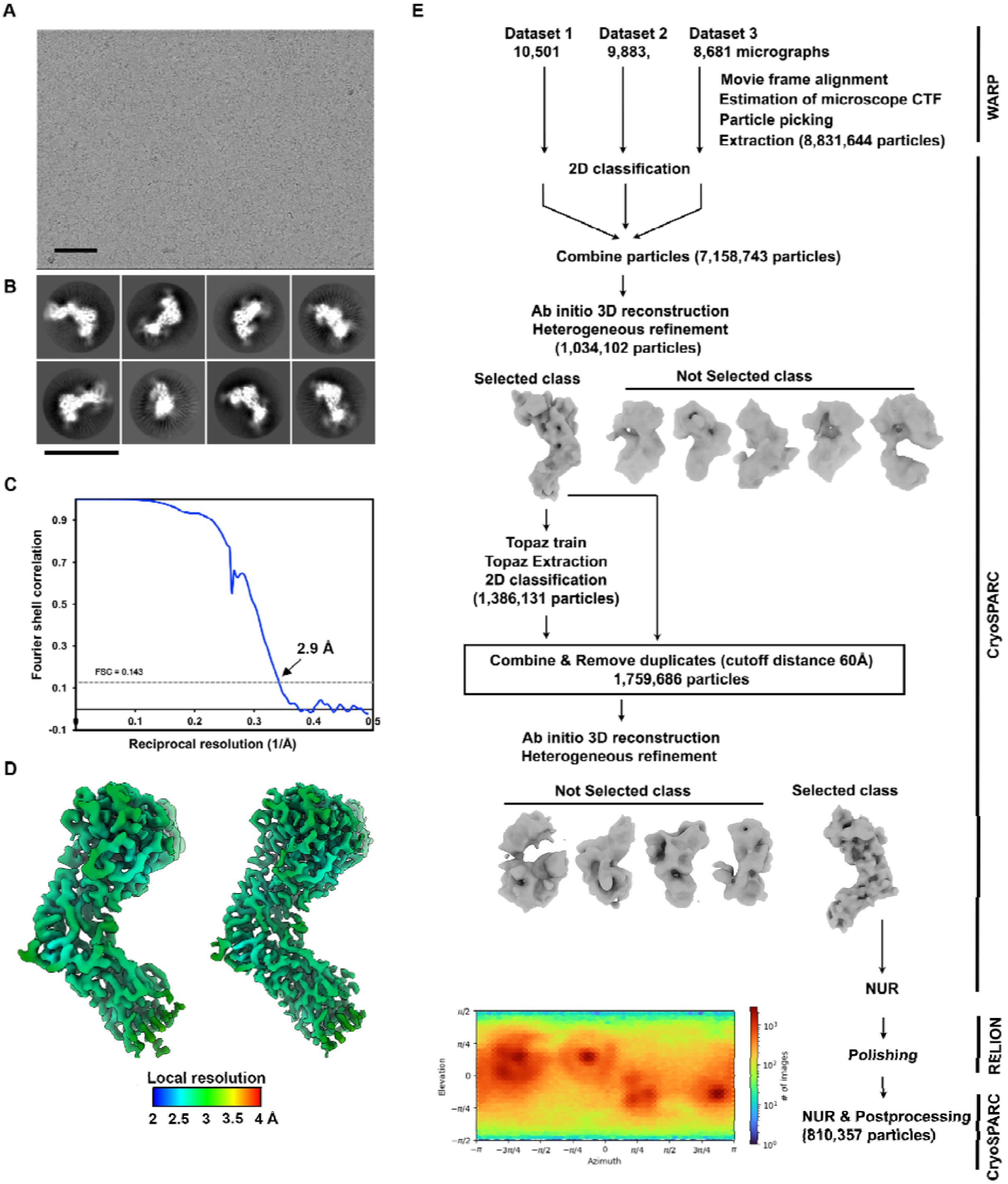

Figure S2. CryoEM data processing of the TMPRSS2-bound HKU1 RBD dataset

(A-B) Representative electron micrograph and 2D class averages of the TMPRSS2-bound HKU1 RBD complex embedded in vitreous ice. The scale bars represent 100 nm and 150Å respectively. (C) Gold-standard Fourier shell correlation curve. The 0.143 cutoff is indicated by a horizontal dashed line. (D) Local resolution estimation calculated using cryoSPARC and plotted on the unsharpened (left) and sharpened (right) maps. (E) Data processing flowchart. CTF: contrast transfer function; NUR: non-uniform refinement. The angular distribution calculated in

bioRxiv preprint doi: https://doi.org/10.1101/2024.01.09.574565; this version posted January 9, 2024. The copyright holder for this preprint (which was not certified by peer review) is the author/funder, who has granted bioRxiv a license to display the preprint in perpetuity. It is made available under aCC-BY 4.0 International license.

cryoSPARC for particle projections is shown. The heat map shows the number of particles for each viewing angle.

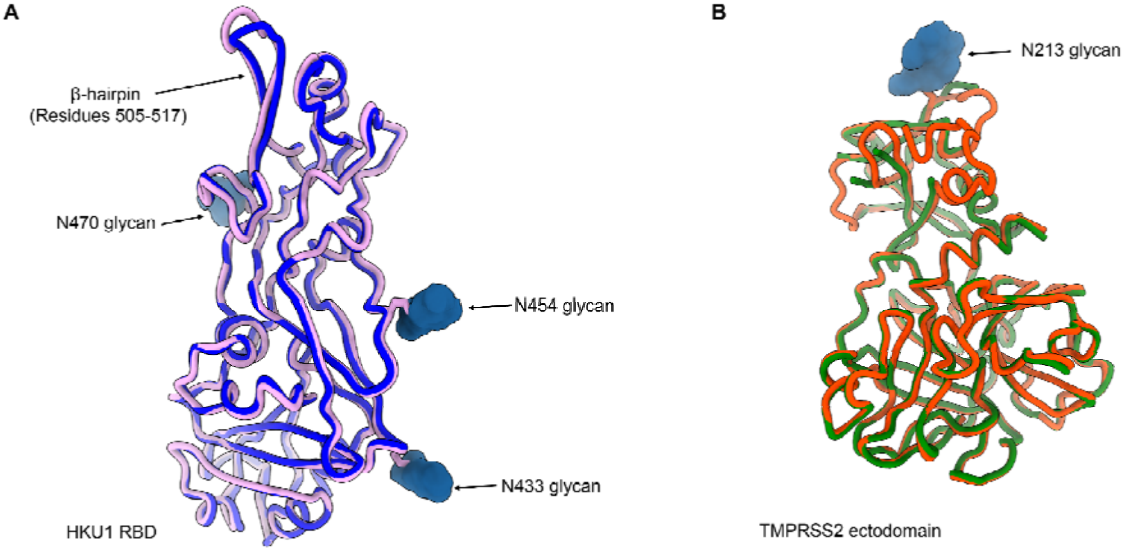

Figure S3. Comparison of the HKU1 RBD and TMPRSS2 structures to the TMPRSS2-bound HKU1 RBD cryoEM structure.

(A) Ribbon diagram of the cryoEM structure of the HKU1 RBD (purple) bound to the human TMPRSS2 ectodomain superimposed to the crystal structure of the apo HKU1 RBD (blue, PDB 5KWB). The TMPRSS2 ectodomain is omitted for clarity. (B) Ribbon diagram of the cryoEM structure of the human TMPRSS2 ectodomain (red) bound to the HKU1 RBD superimposed to the crystal structure of the nafamostat-bound TMPRSS2 (green, PDB 7MEQ). The HKU1 RBD is omitted for clarity.

bioRxiv preprint doi: https://doi.org/10.1101/2024.01.09.574565; this version posted January 9, 2024. The copyright holder for this preprint (which was not certified by peer review) is the author/funder, who has granted bioRxiv a license to display the preprint in perpetuity. It is made available under aCC-BY 4.0 International license.

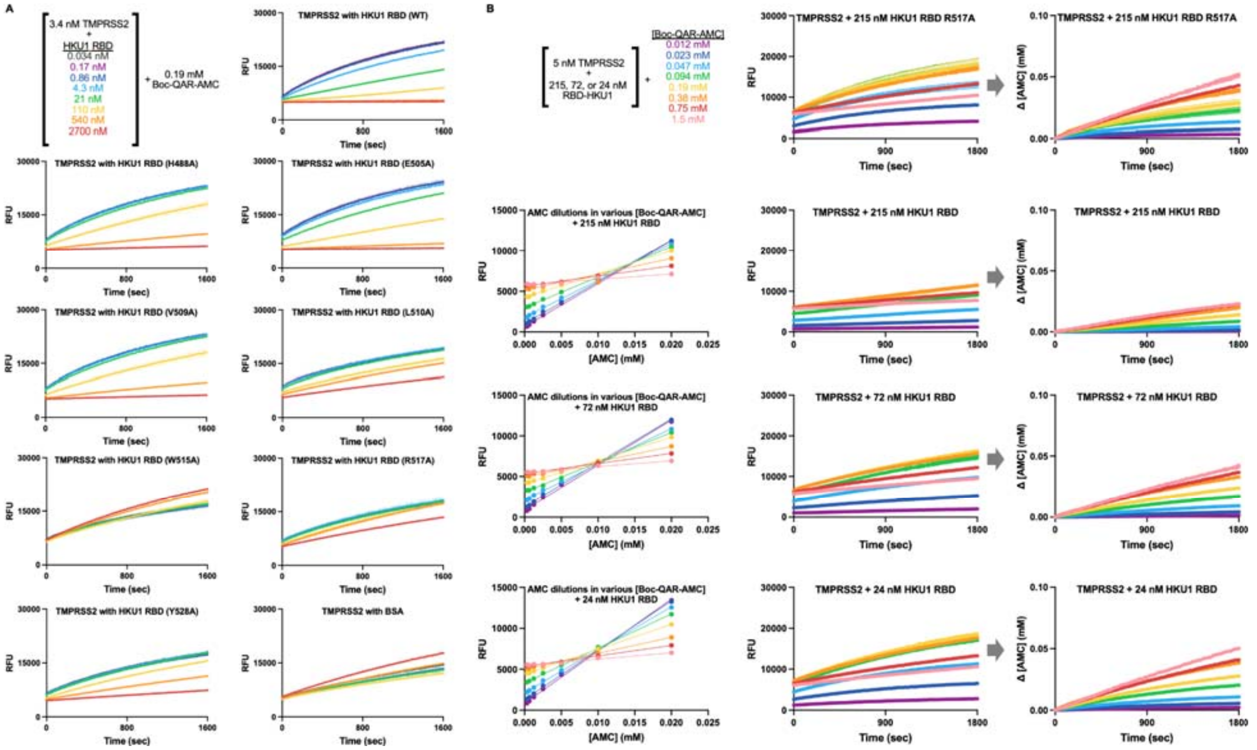

Figure S4. Enzymatic characterization of TMPRSS2 inhibition by the wildtype isolate N1 and alanine interface mutant HKU1 RBDs. (A) Raw data used for Figure 3B; the upper left panel shows the color key used for the other panels. Data show measured fluorescence (RFU) over time with 3.4 nM TMPRSS2 (harboring the N-terminal SUMO fusion, the C379-T447C disulfide bond and the N249 glycan) incubated with 0.19 mM Boc-QAR-AMC at 22ºC in the presence of various concentrations of HKU1 isolate N1 RBD and alanine interface mutant RBDs. (B) Raw data and calculations used for Figure 3C-D; the upper left panel shows the color key used for the other panels. Lower left panels show a summary of AMC standard curves used to calculate AMC release from the Boc-QAR-AMC peptide substrate with 215, 72, and 24 nM HKU1 RBD, accounting for the inner filter effect. The middle panels show measured fluorescence (RFU) over time with 5 nM TMPRSS2 and 215, 72, or 24 nM WT HKU1 RBD, or 215 nM HKU1 R517A RBD, incubated with various concentrations of Boc-QAR-AMC at 22 ºC. The right panel shows the change in calculated AMC concentration over time.

bioRxiv preprint doi: https://doi.org/10.1101/2024.01.09.574565; this version posted January 9, 2024. The copyright holder for this preprint (which was not certified by peer review) is the author/funder, who has granted bioRxiv a license to display the preprint in perpetuity. It is made available under aCC-BY 4.0 International license.

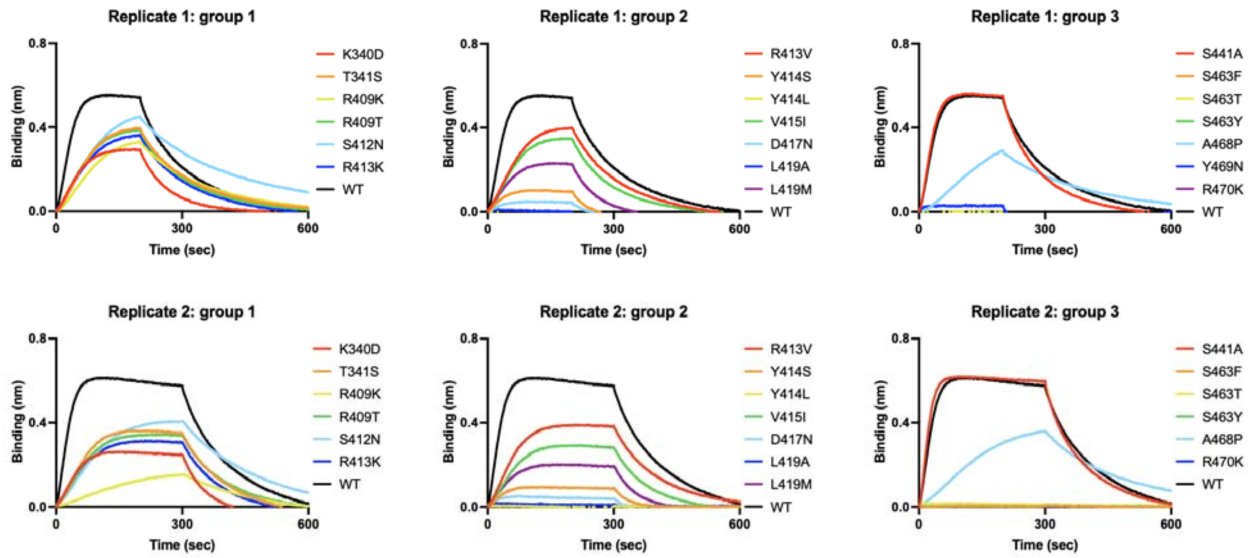

Figure S5. Binding of TMPRSS2 point mutants to the biotinylated HKU1 RBD immobilized on biolayer interferometry SA biosensors. The top and bottom rows show baseline-subtracted response curves for the first and second biological replicates. For the first replicate, biotinylated HKU1 RBD-loaded SA tips were dipped into 100 nM TMPRSS2 for 200 seconds followed by dissociation for 500 seconds. For the second replicate, biotinylated HKU1 isolate N1 RBD-loaded SA tips were dipped into 100 nM TMPRSS2 for 300 seconds followed by dissociation for 500 seconds. To facilitate visualization, the binding curves from point mutants are split into three groups and include wildtype (WT) TMPRSS2 for reference.

bioRxiv preprint doi: https://doi.org/10.1101/2024.01.09.574565; this version posted January 9, 2024. The copyright holder for this preprint (which was not certified by peer review) is the author/funder, who has granted bioRxiv a license to display the preprint in perpetuity. It is made available under aCC-BY 4.0 International license.

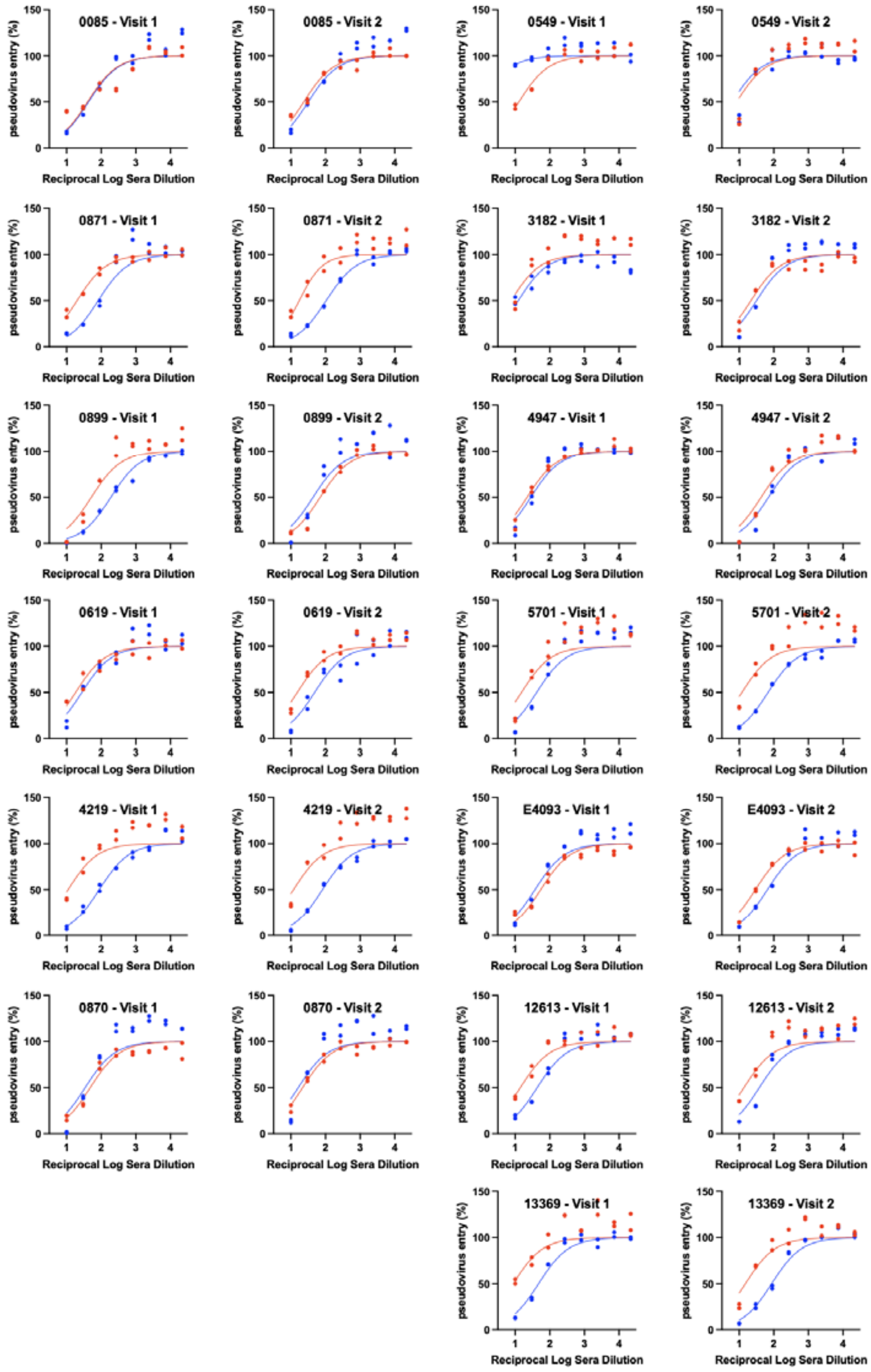

Figure S6. Evaluation of HKU1 infection-elicited serum neutralizing activity in humans. Dose-response curves for neutralization of VSV pseudotyped with wildtype isolate N1 HKU1 S (WT, red) or the N355 glycan knockout (S357A, blue) mutant by human serum antibodies, shown in red and blue, respectively. Visits 1 and 2 correspond to blood draws at the time of PCR-positive testing and 30 days later, respectively[52,53].

bioRxiv preprint doi: https://doi.org/10.1101/2024.01.09.574565; this version posted January 9, 2024. The copyright holder for this preprint (which was not certified by peer review) is the author/funder, who has granted bioRxiv a license to display the preprint in perpetuity. It is made available under aCC-BY 4.0 International license.

Table S1. Amino acid sequences of designed TMPRSS2 constructs.

**Full length TMPRSS2 (S441A) Mus musculus Q3UKE3:**<br>MALNSGSPPGIGPCYENHGYQSEHICPPRPPVAPNGYNLYPAQYYPSPVPQYAPRITTQASTSVIHTHPKSSGALCTSKSKKSLCL<br>ALALGTVLTGAAVAAVLLWRFWDSNCSTSEMECGSSGTCISSSLWCDGVAHCPNGEDENRCVRLYGQSFILQVYSSQRKAWYP<br>VCQDDWSESYGRAACKDMGYKNNFYSSQGIPDQSGATSFMKLNVSSGNVDLYKKLYHSDSCSSRMVVSLRCIECGVRSVKRQS<br>RIVGGLNASPGDWPWQVSLHVQGVHVCGGSIITPEWIVTAAHCVEEPLSSPRYWTAFAGILRQSLMFYGSRHQVEKVISHPNYDS<br>KTKNNDIALMKLQTPLAFNDLVKPVCLPSPGMMLDLDQECWISGWGATYEKGKTSDVLNAAMVPLIEPSKCNSKYIYNNLITPAMIC<br>AGFLQGSVDSCQGDSGGPLVTLKNGIWWLIGDTSWGSGCAKALRPGVYGNVTVFTDWIYKQMRANSSGDYKDHDGDYKDHDID<br>YKDDDDK
**Full length TMPRSS2 (S441A) Ratus Norvegicus Q6P7D7:**<br>MALNSGSPPGIGPYYENHGYQSEHVYSPRPPVSPSGYNLYPAQSCPSPVPQYAPRVTTQASTPAIHIQPRSSGTLCTSKSKKSML<br>VALALGTVLAGAAVAAGLLWKFWDSKCSSSEMECGSSGTCISSSLWCDGVSQCPNGEDENRCVRLYGTSFTLQVYSSQRKAWY<br>PVCQDDWNESYGRAACKDMGYKNSFYSSQGIPDQSGATSFMKLNVSAGNVDLYKKLYHSDSCSSRMVVSLRCIECGVRSVRRQ<br>SRIVGGSTASPGDWPWQVSLHVQGIHVCGGSIITPEWIVTAAHCVEEPLSSPRYWTAFAGILKQSLMFYGSRHQVEKVISHPNYDS<br>KTKNNDIALMKLQTPLAFNDVVKPVCLPNPGMMLDLAQECWISGWGATYEKGKTSDVLNAAMVPLIEPSKCNSKYIYNNLITPAMI<br>CAGFLQGSVDSCQGDSGGPLVTLKNEIWWLIGDTSWGSGCAKAYRPGVYGNVTVFTDWIYQQMRANSSGDYKDHDGDYKDHDI<br>DYKDDDDK
**Full length TMPRSS2 (S441A) Rhinolophus ferrumequinum A0A671DXW4:**<br>MALNSGSPPGVGPYYENHGYQPESLYPPRPAVAPSAYSVYPARYYTPVVPQYTPRVMTHTSTPAIHTQPKSPSGTLCTSKTKKAL<br>CITFSLGAVLVGAVVAAVLLWKFMENKCSVSGIECGSSGTCVSASHWCDGILHCPSGEDENRCVRLYGPNFILQVYSSQRKSWHP<br>VCQDDWSENYGRAACQDMGYRNSFYSSQGIVDDSGATSFMKLNISAGHTDLYKKLYHSDVCSSKTVVSLRCIECGVNTKMSRQS<br>RIVGGTSAALGDWPWQVSLHVQGVHVCGGSIITPEWIVTAAHCVEEPLNNPRYWTAFAGILRQSFMFYGSGYRVEKVISHPNYDS<br>KSKNNDIALMKLQTPLTFNDKVKPVCLPNPGLMLEPTQSCWISGWGATYEKGKTSEVLNAVMVPLIEPWQCNSKYVYNNLITPAMI<br>CAGYLQGTIDSCQGDSGGPLVTLKSSVWWLIGDTSWGSGCAKANRPGVYGNVTVFTDWIYRQMRANSSGDYKDHDGDYKDHDI<br>DYKDDDDK
**Full length TMPRSS2 (S441A) Chlorocebus sabaeusgreen A0A0D9R7A1:**<br>MALNSGSPPGVGPYYENHGYQPENPYPAQPTVAPNVYEVHPAQYYPSPVPQYIPSVLTHASNPAVRTQPKSPSGTVCTSKTKKA<br>LCVTMTLGAVLVGAALAAGLLWKFMGSKCSDSGIECDSSGTCISSSNWCDGVSHCPSGEDENRCVRLYGPNFILQVYSSQRKSW<br>HPVCRDDWNENYARAACRDMGYQNRFYSSQGIADNSGATSFMKLNTSAGNVDIYKKLYHSDACSSKAVVSLRCIACGVRSNLSR<br>QSRIVGGQNALPGAWPWQVSLHVQNIHVCGGSIITPEWIVTAAHCVEKPLNSPWQWTAFAGILTQSSMFYVKGHRVEKVISHPNY<br>DSKTKNNDIALMKLHTPLTFNELVKPVCLPNPGMMLEPEQHCWISGWGATQEKGKTSDMLNAAMVPLIEPRRCNSKRVYDGLVT<br>PAMICAGFLQGTVDSCQGDSGGPLVTLKNDVWWLIGDTSWGSGCAQANRPGVYGNVTVFTDWIYRQMRADDSGDYKDHDGDY<br>KDHDIDYKDDDDK
**Full length TMPRSS2 (S441A) Mesocricetus auratus hamster A0A1U8C7X1:**<br>MALNSGSPPGIGPYYENHGFQSEHIYPPRPPVAPDVYNPYPPQNYPPPVPQYFPRVTTQASTTVTHTQPHSSGKLCTSTSKTKKS<br>LCFALSLGIVLVGAAVAAVLLWKFLPGCSTSEMECMSSGTCISSSLWCDGTSHCPNGEDENRCVRLYGPSFTLQVYSSQRKAWY<br>PVCQDDWNDSYGRAACKDMGYKNNFYYTQGIPDSSGATSFMKLNISAGNIDLYKKLYHSDSCSSRMVVSLRCIQCGVRSATRQS<br>RIVGGSNASPGDWPWQVSLHVQGVHVCGGSIITPEWIVTAAHCVEEPLNSPRYWTAFAGILSQSLMFYGSRHQVEKVISHPNYNS<br>ETKNNDIALMKLQTPLTFNDLVKPVCLPNPGMMLDPAQECWISGWGSTYEKGKTSDMLNAAMVPLIERSKCNNKYIYNNLITPAMV<br>CAGFLQGTVDSCQGDSGGPLVTLKNDIWWLIGDTSWGSGCAKALRPGVYGNVTVFTDWIYQQMMANSSGDYKDHDGDYKDHDI<br>DYKDDDDK
**Full length TMPRSS2 (S441A) Mustela furo ferret A0A8U0SMZ2:**<br>MALNAGSPPGVGPYYENHGYQPESLYPAPPATVPSVYVAYPAAYYPPAVPQYTPRVLTQASTPAVRTQPKSPSGTACTAKAKKA<br>LCITISLGAVLAGAAVTAVLLWKFMENKCSVSGIECGSSGTCISPSHWCDGVLHCPSGEDENRCVRLYGPNFILQVYSAQRKSWH<br>PVCQDDWSDSYGRAACQDMGYRNSFYSSQGIVDDSGASSFMKLNTSAGNTDLYKKLYHSDICASKTVVSLRCIECGVAGKTMRQ<br>SRIVGGSSASPGDWPWQVSLHVQGTHVCGGSIITPEWIVTAAHCVEEPLNNPRYWTVFAGVLRQSFMFYGHGYRVGKVISHPSY<br>DSKTKNNDIALMKLQTPLTFSDKVKPVCLPNPGMMLEPNQSCWISGWGATHEKGKTSDELNAVMVPLIEPWRCNSKYVYNSLVT<br>PAMICAGYLRGGTDSCQGDSGGPLVTLKSRIWWLIGDTSWGSGCAKANRPGVYGNVTVFTDWIYRQMRANSSGDYKDHDGDYK<br>DHDIDYKDDDDK
**Full length TMPRSS2 (S441A) Camelus dromedarius XP_010993167.1:**<br>MALNSGSPPGVGPYYENHGYQPESFYPLKPSAASSAYMVYPAQYYPPAVPQYTPRVQTHTSTPVIRMQPKPPSETVCTSKTKKV<br>LCVTLGLGAILVGAVLAAVLLWKFKESRCSASEMECGSSGTCISPSQWCDGVLHCPGGEDENQCVRLYGPNFILQVYSPQRKSW

bioRxiv preprint doi: https://doi.org/10.1101/2024.01.09.574565; this version posted January 9, 2024. The copyright holder for this preprint (which was not certified by peer review) is the author/funder, who has granted bioRxiv a license to display the preprint in perpetuity. It is made available under aCC-BY 4.0 International license.

HPVCQEDWSESFGRAMCQDLGYGNSFYSSQGVVDDSGATSFMKLNISANNIDLYKKLYHSDVCSSKRVVSLRCIECGVSEKTSR
QSRIVGGSSANLGDWPWQVSLHVQGIHVCGGSIITPEWIVTAAHCVEQPLSNAKIWTAFAGILSQSLMIYGNGYQIAKVISHPNYDS
KTKNNDIALMKLQTPLTFNDRVKPVCLPNPGMMLEATQSCWISGWGATYEKGKTSDVLNAVMVHLIEPWKCNSKYVYNNLITSAM
ICAGYLQGGVDSCQGDSGGPLVTLKNSVWWLLGDTSWGSGCAKAYRPGVYGNMTVFTDWIYRQMRANSSGDYKDHDGDYKD
HDIDYKDDDDK

Full length TMPRSS2 (S441A) Gallus domesticus XP_015156666.1:
MTSTVNPPPYYENHGFQTENYYSARPQVGANPYPQYFSTNVPSVPTYIPRVSTHQSSIPVAPPSSSSRMCSSSIKKIVIITLSILLVIC
CAIAAFLIWYFVENRCLGSLIECGSSGVCISPSVWCDGVTDCPNGEDENRCVRLYGPNFILEVYSPVSQTWYPVCQDDWTDDFGK
IACEDMGYNVDTYYYSQGVAAEVSFKSFMKLNTSAGNTDLYKRLQSSDYCASGNVVSLRCIECGLPTKSTAVMSRIVGGSMASLG
QWPWQVSLHVQDTHVCGGSIITREWLVTAAHCVEGLFSDPYIWSVYAGILSQNEMHSRPGYRVQKIISHPNYDTDSKDNDVALMK
LETPLSFTNTIRPVCLPNPGMMFQPNQQCWISGWGAEYQGGKTANDLNYVMVPLIERSTCNSVYVYDGMVLPTMVCAGYLQGGI
DSCQGDSGGPLVTNKNSVWWLVGDTSWGTGCASPNRPGVYGNMTVFTDWIYKNMQANRSGDYKDHDGDYKDHDIDYKDDDD
K

Full length TMPRSS2 (S441A) Chamaeleo jacksonii G1KE28:
MNSRPPPYYENYAYQPENIAPPRHAGGYMYPPYPSPYYPSSVPHYIPRVSTNQSTPVPTVVQPKATPAKCMPKTRKAVCLFLAIS
VLLIGGAIAAVLIWHFVTDSCFGSKIKCGTTGMCVAPSQWCDGIRDCPNNEDETRCVRLFGPEFQLEVYSSESKDWYPVCSDDWN
DKHGKTACEDLGYNSNTYFKSQTIPLMSASKGFMKLNASAGDIDLYKKLYNSKSCSSRSLVSLQCIDCGTRRVNRRNRIVGGTSAS
LGDWPWQVSLHSSGTHLCGGSIITPEWIVTAAHCVEKAFSNPNYWTVFAGILTQPEMISSKGHKVAKVIPHPGYDTSSKTNDVALM
KLQSPLVFDEFVRPVCLPNPGMMFQSDQPYWISGWGAVEQKGPTSKKLNAARIRLIDSDTCNNRYIYNGLILPTMICAGYLNGGID
SCQGDSGGPLVTSKDSLWWLVGDTSWGTGCATKYRPGVYGNMTVFTDWIYKNMQANRRSGDYKDHDGDYKDHDIDYKDDDD
K

Full length TMPRSS2 (S441A) Xenopus laevis XP_018104413.1:
MAGRDYRWKSPSPPYFENYGFQQDNNNAFSRPVQPNFYEAFQPPRPQLSPAPHYIPQVSTIHSVPAINEHKSQTWWTPRRKKIA
CIVAATSVLIALLIVGAVLCWYFVTMSCQMKCGTSGSCVRSIQWCDGVAQCPGGEDESYCVRMYGPDFQLQAYIPATSSWLSVCN
ENWGDSQGRSVCQDMGYSTYVKSSSVSATSATEGYLKLNTSVVNGKLQSRIYKSSFCTSGVVTLRCIECGSSTKNVENRIVGGS
QASLGDWPWQVSLQYNERHVCGGSIITSNYILTAAHCVEGAYSSPYAWTVYVGSISRSTAGIRYYVKSVIGHQKYDTKTKNNDVAL
MRLKISILFSSTVQPVCLPNAGMPWASGQSCWTSGWGATYEGGTSSNVLNAAMVPLIDADTCNRPAVYNGAVTSTMICAGYLRG
GIDSCQGDSGGPLVTKTNSLWWLVGDTSWGTGCANVNKPGVYGNITEFLPWIFLQMQTYGSGDYKDHDGDYKDHDIDYKDDDD
K

Full length TMPRSS2 (S441) Homo sapiens:
MALNSGSPPAIGPYYENHGYQPENPYPAQPTVVPTVYEVHPAQYYPSPVPQYAPRVLTQASNPVVCTQPKSPSGTVCTSKTKKA
LCITLTLGTFLVGAALAAGLLWKFMGSKCSNSGIECDSSGTCINPSNWCDGVSHCPGGEDENRCVRLYGPNFILQVYSSQRKSWH
PVCQDDWNENYGRAACRDMGYKNNFYSSQGIVDDSGSTSFMKLNTSAGNVDIYKKLYHSDACSSKAVVSLRCIACGVNLNSSRQ
SRIVGGESALPGAWPWQVSLHVQNVHVCGGSIITPEWIVTAAHCVEKPLNNPWHWTAFAGILRQSFMFYGAGYQVEKVISHPNYD
SKTKNNDIALMKLQKPLTFNDLVKPVCLPNPGMMLQPEQLCWISGWGATEEKGKTSEVLNAAKVLLIETQRCNSRYVYDNLITPAM
ICAGFLQGNVDSCQGDSGGPLVTSKNNIWWLIGDTSWGSGCAKAYRPGVYGNVMVFTDWIYRQMRADG

TMPRSS2 ectodomain (S441A):
MTRLTVLALLAGLLASSRASMGSKCSNSGIECDSSGTCINPSNWCDGVSHCPGGEDENRCVRLYGPNFILQVYSSQRKSWHPVC
QDDWNENYGRAACRDMGYKNNFYSSQGIVDDSGSTSFMKLNTSAGNVDIYKKLYHSDACSSKAVVSLRCIACGVNLNDDDDKIV
GGESALPGAWPWQVSLHVQNVHVCGGSIITPEWIVTAAHCVEKPLNNPWHWTAFAGILRQSFMFYGAGYQVEKVISHPNYDSKT
KNNDIALMKLQKPLTFNDLVKPVCLPNPGMMLQPEQLCWISGWGATEEKGKTSEVLNAAKVLLIETQRCNSRYVYDNLITPAMICA
GFLQGNVDSCQGDAGGPLVTSKNNIWWLIGDTSWGSGCAKAYRPGVYGNVMVFTDWIYRQMRADGDDDDKSGHHHHHHHH

TMPRSS2 ectodomain (S441A + DS):
MTRLTVLALLAGLLASSRASMGSKCSNSGIECDSSGTCINPSNWCDGVSHCPGGEDENRCVRLYGPNFILQVYSSQRKSWHPVC
QDDWNENYGRAACRDMGYKNNFYSSQGIVDDSGSTSFMKLNTSAGNVDIYKKLYHSDACSSKAVVSLRCIACGVNLNDDDDKIV
GGESALPGAWPWQVSLHVQNVHVCGGSIITPEWIVTAAHCVEKPLNNPWHWTAFAGILRQSFMFYGAGYQVEKVISHPNYDSKT
KNNDIALMKLQKPLTFNDLVKPVCLPNPGMMLQPEQLCWISGWGATEEKGKTSEVLNAAKVLLIETQRCNSRYVYDNLITPAMICA
GFLQGNVDSCQGDAGGPLVCSKNNIWWLIGDTSWGSGCAKAYRPGVYGNVMVFTDWIYRQMRADGDDDDKSGHHHHHHHH

TMPRSS2 ectodomain (S441 + DS):
MTRLTVLALLAGLLASSRASMGSKCSNSGIECDSSGTCINPSNWCDGVSHCPGGEDENRCVRLYGPNFILQVYSSQRKSWHPVC
QDDWNENYGRAACRDMGYKNNFYSSQGIVDDSGSTSFMKLNTSAGNVDIYKKLYHSDACSSKAVVSLRCIACGVNLNDDDDKIV
GGESALPGAWPWQVSLHVQNVHVCGGSIITPEWIVTAAHCVEKPLNNPWHWTAFAGILRQSFMFYGAGYQVEKVISHPNYDSKT

bioRxiv preprint doi: https://doi.org/10.1101/2024.01.09.574565; this version posted January 9, 2024. The copyright holder for this preprint (which was not certified by peer review) is the author/funder, who has granted bioRxiv a license to display the preprint in perpetuity. It is made available under aCC-BY 4.0 International license.

KNNDIALMKLQKPLTFNDLVKPVCLPNPGMMLQPEQLCWISGWGATEEKGKTSEVLNAAKVLLIETQRCNSRYVYDNLITPAMICA
GFLQGNVDSCQGDSGGPLVCSKNNIWWLIGDTSWGSGCAKAYRPGVYGNVMVFTDWIYRQMRADGDDDDKSGHHHHHHHH

TMPRSS2 ectodomain (S441A + DS + SUMO):
MTRLTVLALLAGLLASSRASMSDSEVNQEAKPEVKPEVKPETHINLKVSDGSSEIFFKIKKTTPLRRLMEAFAKRQGKEMDSLRFLY
DGIRIQADQTPEDLDMEDNDIIEAHRENDDDDKTTGSKCSNSGIECDSSGTCINPSNWCDGVSHCPGGEDENRCVRLYGPNFILQ
VYSSQRKSWHPVCQDDWNENYGRAACRDMGYKNNFYSSQGIVDDSGSTSFMKLNTSAGNVDIYKKLYHSDACSSKAVVSLRCI
ACGVNLNDDDDKIVGGESALPGAWPWQVSLHVQNVHVCGGSIITPEWIVTAAHCVEKPLNNPWHWTAFAGILRQSFMFYGAGYQ
VEKVISHPNYDSKTKNNDIALMKLQKPLTFNDLVKPVCLPNPGMMLQPEQLCWISGWGATEEKGKTSEVLNAAKVLLIETQRCNSR
YVYDNLITPAMICAGFLQGNVDSCQGDAGGPLVCSKNNIWWLIGDTSWGSGCAKAYRPGVYGNVMVFTDWIYRQMRADGDDDD
KSGHHHHHHHH

TMPRSS2 ectodomain (S441 + DS + SUMO):
MTRLTVLALLAGLLASSRASMSDSEVNQEAKPEVKPEVKPETHINLKVSDGSSEIFFKIKKTTPLRRLMEAFAKRQGKEMDSLRFLY
DGIRIQADQTPEDLDMEDNDIIEAHRENDDDDKTTGSKCSNSGIECDSSGTCINPSNWCDGVSHCPGGEDENRCVRLYGPNFILQ
VYSSQRKSWHPVCQDDWNENYGRAACRDMGYKNNFYSSQGIVDDSGSTSFMKLNTSAGNVDIYKKLYHSDACSSKAVVSLRCI
ACGVNLNDDDDKIVGGESALPGAWPWQVSLHVQNVHVCGGSIITPEWIVTAAHCVEKPLNNPWHWTAFAGILRQSFMFYGAGYQ
VEKVISHPNYDSKTKNNDIALMKLQKPLTFNDLVKPVCLPNPGMMLQPEQLCWISGWGATEEKGKTSEVLNAAKVLLIETQRCNSR
YVYDNLITPAMICAGFLQGNVDSCQGDSGGPLVCSKNNIWWLIGDTSWGSGCAKAYRPGVYGNVMVFTDWIYRQMRADGDDDD
KSGHHHHHHHH

TMPRSS2 ectodomain (S441A + DS + SUMO + N249 glycan):
MTRLTVLALLAGLLASSRASMSDSEVNQEAKPEVKPEVKPETHINLKVSDGSSEIFFKIKKTTPLRRLMEAFAKRQGKEMDSLRFLY
DGIRIQADQTPEDLDMEDNDIIEAHRENDDDDKTTGSKCSNSGIECDSSGTCINPSNWCDGVSHCPGGEDENRCVRLYGPNFILQ
VYSSQRKSWHPVCQDDWNENYGRAACRDMGYKNNFYSSQGIVDDSGSTSFMKLNTSAGNVDIYKKLYHSDACSSKAVVSLRCI
ACGVNLNSSDDDDKIVGGESALPGAWPWQVSLHVQNVHVCGGSIITPEWIVTAAHCVEKPLNNPWHWTAFAGILRQSFMFYGAG
YQVEKVISHPNYDSKTKNNDIALMKLQKPLTFNDLVKPVCLPNPGMMLQPEQLCWISGWGATEEKGKTSEVLNAAKVLLIETQRCN
SRYVYDNLITPAMICAGFLQGNVDSCQGDAGGPLVCSKNNIWWLIGDTSWGSGCAKAYRPGVYGNVMVFTDWIYRQMRADGDD
DDKSGHHHHHHHH

TMPRSS2 ectodomain (S441 + DS + SUMO + N249 glycan):
MTRLTVLALLAGLLASSRASMSDSEVNQEAKPEVKPEVKPETHINLKVSDGSSEIFFKIKKTTPLRRLMEAFAKRQGKEMDSLRFLY
DGIRIQADQTPEDLDMEDNDIIEAHRENDDDDKTTGSKCSNSGIECDSSGTCINPSNWCDGVSHCPGGEDENRCVRLYGPNFILQ
VYSSQRKSWHPVCQDDWNENYGRAACRDMGYKNNFYSSQGIVDDSGSTSFMKLNTSAGNVDIYKKLYHSDACSSKAVVSLRCI
ACGVNLNSSDDDDKIVGGESALPGAWPWQVSLHVQNVHVCGGSIITPEWIVTAAHCVEKPLNNPWHWTAFAGILRQSFMFYGAG
YQVEKVISHPNYDSKTKNNDIALMKLQKPLTFNDLVKPVCLPNPGMMLQPEQLCWISGWGATEEKGKTSEVLNAAKVLLIETQRCN
SRYVYDNLITPAMICAGFLQGNVDSCQGDSGGPLVCSKNNIWWLIGDTSWGSGCAKAYRPGVYGNVMVFTDWIYRQMRADGSG
LNDIFEAQKIEWHEQSGHHHHHHHH

bioRxiv preprint doi: https://doi.org/10.1101/2024.01.09.574565; this version posted January 9, 2024. The copyright holder for this preprint (which was not certified by peer review) is the author/funder, who has granted bioRxiv a license to display the preprint in perpetuity. It is made available under aCC-BY 4.0 International license.

Table S2. Cryo-EM data collection, refinement and validation statistics

	HKU1 RBD - TMPRSS2<br>PDB 8VGT<br>EMD-43224
**Data collection and processing**	
Magnification	105,000
Voltage (kV)	300
Electron exposure (e–/Å^2)	60
Defocus range (μm)	-0.2 - -3.5
Pixel size (Å)	0.835
Symmetry imposed	C1
Initial particle images (no.)	8,831,644
Final particle images (no.)	810,357
Map resolution (Å)	2.9
FSC threshold	0.143
**Refinement**	
Model resolution (Å)	3.1
FSC threshold	0.5
Map sharpening *B* factor (Å^2)	-130
Model composition	
Non-hydrogen atoms	5029
Protein residues	637
Ligands	5
B factors (Å^2)	
Protein	16.34
Ligand	28.76
R.m.s. deviations	
Bond lengths (Å)	0.011
Bond angles (°)	1.219
**Validation**	
MolProbity score	1.06
Clashscore	0.82
Poor rotamers (%)	0.71
Ramachandran plot	
Favored (%)	95.56
Allowed (%)	4.12
Disallowed (%)	0.32

Data S1. Human TMPRSS2 SNPs at the HKU1 RBD-interacting site present in the GnomADv4 database.

Data S2. Human TMPRSS2 SNPs at the HKU1 RBD-interacting site present in the Regeneron Million Exome Variant database.

bioRxiv preprint doi: https://doi.org/10.1101/2024.01.09.574565; this version posted January 9, 2024. The copyright holder for this preprint (which was not certified by peer review) is the author/funder, who has granted bioRxiv a license to display the preprint in perpetuity. It is made available under aCC-BY 4.0 International license.

Data S3. Demographic data for HKU1 infected serum donors

Manuscript code	Country of isolation	Age	Gender	Collection date V1	Collection date V2
HK-01	Netherlands	47	2	2/3/10	3/5/10
HK-02	Spain	36	2	3/16/10	4/20/10
HK-03	Spain	27	2	3/5/10	4/26/10
HK-04	Spain	38	2	4/8/10	5/12/10
HK-05	Poland	56	1	3/8/10	4/16/10
HK-06	Poland	54	2	1/21/10	2/19/10
HK-07	Poland	48	1	3/3/10	4/2/10
HK-08	Poland	28	2	2/23/10	4/19/10
HK-09	Poland	35	1	3/4/10	4/26/10
HK-10	Poland	47	1	2/10/10	3/15/10
HK-11	Poland	26	2	2/8/10	3/11/10
HK-12	United Kingdom	53	2	12/11/07	1/18/08
HK-13	Spain	27	2	2/16/09	4/16/09
